# Medical Societies and Medical Journals

**Published:** 1875-09

**Authors:** 


*Medical Societies and Medical Journals.*

The value of good healthy Medical Societies and Medical Journals to the practitioner of medicine, is not easily overestimated. One who is in the habit of frequent attendance upon the one, and of taking an active part in the discussion of the presented propositions, and of taking and reading the other, has only to rub against a fellow practitioner who does neither, to learn how much he is in advance of his fellows. There is nothing in medicine more nearly true, than that "Journal readers are the professional leaders," and where this is combined with practical attendance upon good medical societies, where the meetings are frequent and the discussions practical and scientific, there can be no doubt of where the leadership will rest.

I was much amused a few days since, while listening to a conversation that occurred between two physicians on the advancements made by the medical professiou in the last twenty years. One of them was, to all intents and purposes, a journal reader and a society attender; while the other neither reads journals nor attends societies. The latter allowed that there was no advancement worthy of note in any branch of medicine in the last twenty years, except in surgery, but that we gave the same medicine, in the same doses, for the same diseases, that we did two decades ago. The former at once took issue and pointed out many points of notable improvement, other than those in surgery, but, unfortunately, his listener was not even able to appreciate the points made, for the reason that he was so far in the dark that he could not comprehend them. The difference in the success and safety of such men in practice is not only apparent to medical men, but also to all intelligent people. It is not absolutely necessary that a man read many journals in order to be professionally intelligent or skillful, for he may secure the latest and most valuable text-books and succeed well. But even if he does this to perfection, there are thousands of valuable practical and scientific items that will escape his attention for the time, most of them for all time to come. The most advanced text-books are years behind the van of the profession, and the medical journals are at least ten years in advance of the text-books. Hence the careful journal reader will be found far ahead of the text-books. The latter are, how ever, as necessary as the journals, for they give the solid basis on which professional intelligence rests. It is a valid objection often offered against journals that they contain much that is unreliable and trashy. But, while this is true, it cannot be denied that they contain a very great amount of useful and reliable practical and scientific information that can be had nowhere else, and are thus like the medical societies, and, with them, constitute a most effective drill in medicine. No proposition in any branch of medicine will strike two medical men precisely alike, and a point that is overlooked by one will be taken up by another. These varied phases of any proposition are viewed under healthy society discussions, and the pens of the numerous contributors to the journals. Many of these phases are entirely left out of the very best text-books now or ever extant. Beyond this advantage, there is that of obtaining the histories of unique and anomalous cases that cannot possibly be crowded into text-books.

These are but a few of the reasons why every man, who wishes to keep even with, or surpass his professional brethren, should not only consult his text-books often, but also take several of the best medical periodicals possible for him to obtain.

